# N-glycosylation of ICAM-2 is required for ICAM-2-mediated complete suppression of metastatic potential of SK-N-AS neuroblastoma cells

**DOI:** 10.1186/1471-2407-13-261

**Published:** 2013-05-28

**Authors:** Joseph M Feduska, Patrick L Garcia, Stephanie B Brennan, Su Bu, Leona N Council, Karina J Yoon

**Affiliations:** 1 Department of Pharmacology and Toxicology, University of Alabama at Birmingham, Birmingham, AL, USA; 2 Department of Pathology, Division of Anatomic Pathology, University of Alabama at Birmingham, Birmingham, AL, USA

**Keywords:** ICAM-2, CD102, N-glycosylation, Neuroblastoma, Metastasis, Cell motility

## Abstract

**Background:**

Cell adhesion molecules (CAMs) are expressed ubiquitously. Each of the four families of CAMs is comprised of glycosylated, membrane-bound proteins that participate in multiple cellular processes including cell-cell communication, cell motility, inside-out and outside-in signaling, tumorigenesis, angiogenesis and metastasis. Intercellular adhesion molecule-2 (ICAM-2), a member of the immunoglobulin superfamily of CAMs, has six N-linked glycosylation sites at amino acids (asparagines) 47, 82, 105, 153, 178 and 187. Recently, we demonstrated a previously unknown function for ICAM-2 in tumor cells. We showed that ICAM-2 suppressed neuroblastoma cell motility and growth in soft agar, and induced a juxtamembrane distribution of F-actin *in vitro*. We also showed that ICAM-2 completely suppressed development of disseminated tumors *in vivo* in a murine model of metastatic NB. These effects of ICAM-2 on NB cell phenotype *in vitro* and *in vivo* depended on the interaction of ICAM-2 with the cytoskeletal linker protein α-actinin. Interestingly, ICAM-2 did not suppress subcutaneous growth of tumors in mice, suggesting that ICAM-2 affects the metastatic but not the tumorigenic potential of NB cells. The goal of the study presented here was to determine if the glycosylation status of ICAM-2 influenced its function in neuroblastoma cells.

**Methods:**

Because it is well documented that glycosylation facilitates essential steps in tumor progression and metastasis, we investigated whether the glycosylation status of ICAM-2 affected the phenotype of NB cells. We used site-directed mutagenesis to express hypo- or non-glycosylated variants of ICAM-2, by substituting alanine for asparagine at glycosylation sites, and compared the impact of each variant on NB cell motility, anchorage-independent growth, interaction with intracellular proteins, effect on F-actin distribution and metastatic potential *in vivo*.

**Results:**

The *in vitro* and *in vivo* phenotypes of cells expressing glycosylation site variants differed from cells expressing fully-glycosylated ICAM-2 or no ICAM-2. Most striking was the finding that mice injected intravenously with NB cells expressing glycosylation site variants survived longer (P ≤ 0.002) than mice receiving SK-N-AS cells with undetectable ICAM-2. However, unlike fully-glycosylated ICAM-2, glycosylation site variants did not completely suppress disseminated tumor development.

**Conclusions:**

Reduced glycosylation of ICAM-2 significantly attenuated, but did not abolish, its ability to suppress metastatic properties of NB cells.

## Background

Cell adhesion molecules (CAMs) participate in diverse functions in normal cells and tumor cells. These functions include cell-cell and cell-matrix adhesion, migration, oncogenesis, metastasis, tumor suppression or progression, and signal transduction [1-5]. Based on known functions, four major families of CAMs have been identified: integrins, cadherins, selectins, and immunoglobulin (Ig) superfamily. CAMs of the Ig superfamily contain one or more domains similar to the C2 type domains characteristic of a subset of Ig molecules (immunoglobulin-like C2 domain [IgLD]) [6].

Intercellular adhesion molecule-2 (ICAM-2), a 55-60 kDa transmembrane glycoprotein, belongs to the Ig superfamily of CAMs [7,8]. In normal tissue, neovascular endothelial cells express the highest level of ICAM-2. Established vasculature and some leukocytes express a relatively low level of ICAM-2. Functionally, the extracellular domain (ED) of ICAM-2 mediates binding of ICAM-2 on vascular endothelial cells to β_2_-integrins on the surface of leukocytes, an initial step in immune responses [9,10]. ICAM-2 also facilitates neutrophil binding to and migration through vascular endothelium as a component of immune reactions [11,12]. In tumor cells, we recently identified a unique role for ICAM-2 when we demonstrated that ICAM-2 suppressed the metastatic phenotype neuroblastoma (NB) cells [13].

**Figure 1:**
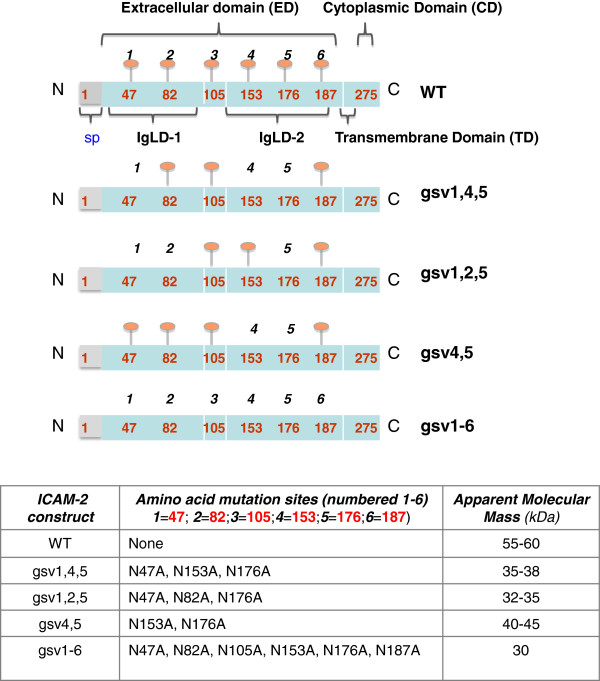
**Schematic structure of ICAM-2 and ICAM-2 variants.** Schematic representation of the structures of ICAM-2 and hypo-glycosylated ICAM-2 glycosylation site variants (gsv). ICAM-2 wild type (WT) contains six glycosylated amino acids in its extracellular domain (ED), at residues 47, 82, 105, 153, 176 and 187. The ED also contains two immunoglobulin-like domains (designated IgLD-1 and -2). Short transmembrane (TD) and cytoplasmic (CD) domains are also present. The N-terminus of the nascent protein contains a 21-amino acid signal peptide (sp) that is not present in the 254 amino acid mature protein. Variants were constructed by substituting alanines (A) for asparagines (N) at the indicated amino acid residues. For example, gsv1,4,5 contains alanines at mutation sites 1, 4, and 5 (amino acids 47, 153, and 176); therefore, gsv1,4,5 is not able to be glycosylated at these three sites. Variant gsv1-6 has alanines at all six glycosylation sites, and is non-glycosylated when expressed in SK-N-AS cell transfectants.

Structurally, ICAM-2 wild type (WT) protein in normal cells [14] and NB cells is identical (Figure 1). ICAM-2 WT is expressed as a 275-amino acid protein containing two Ig-like domains (IgLD-1 and IgLD-2) in the extracellular domain (ED) at the N-terminus of the protein, a 26-amino acid transmembrane domain (TD), and a 26-amino acid cytoplasmic domain (CD). The first 21 amino acids comprise a signal peptide (sp) that routes the nascent protein through the endoplasmic reticulum, for post-translational processing. Processing includes deletion of the sp, to yield a mature protein of 254 amino acids. The mature WT protein consists of a 202 amino acid ED, and the TD and CD noted above. The CD binds to actin cytoskeletal linker ERM proteins (ezrin, radixin, moesin) and to α-actinin [15,16]. The functional significance of the interactions of ICAM-2 with actin linker proteins in tumor cells is largely unknown, with the exception of our recent observation that the interaction of ICAM-2 with α-actinin is essential to ICAM-2-mediated suppression of the metastatic potential of NB cells [13].

The ED of ICAM-2 contains six N-glycosylation sites (asparagines [Asn] residues; amino acids # 47, 82, 105, 153, 178 and 187) [17], a frequent post translational modification of CAM proteins and a modification known, in general, to influence CAM structure and function and to impact the phenotype of tumor cells [18-20]. For example, altered N-glycosylation of β_1_-integrin decreases cell migration [21], and aberrant N-glycosylation of E-cadherin increases discohesion of oral cancer cells [22]. The effect of glycosylation of ICAM-2 on tumor cell function is unknown.

Our previous studies, however, document that fully glycosylated ICAM-2 suppresses the development of disseminated tumors in a murine model of metastatic NB. Importantly, and consistent with preclinical data, we also showed that primary human NB cells that express endogenous ICAM-2 have morphologic and histologic properties of NB cells recognized clinically to have limited metastatic potential [13]. These observations suggest a functional role for ICAM-2 in primary tumor cells. Therefore, in order to identify domains of ICAM-2 that contribute significantly to its function in tumor cells, we generated four ICAM-2 constructs, encoding glycosylation site variants (gsv) of this protein. We expressed each construct in SK-N-AS NB cells, which have no detectable endogenous ICAM-2 and which display a metastatic phenotype in *in vitro* assays and *in vivo* models. We altered the glycosylation status of ICAM-2 by substitution of alanines for asparagines, to prevent glycosylation at specific residues that comprise N-linked glycosylation sites *in situ;* and then determined whether substitutions modulated the ability of ICAM-2 to suppress metastatic properties of NB cells. The data show that hypo-glycosylated variants of ICAM-2 have a significant impact on NB cell phenotype, but to a lesser extent than that seen with ICAM-2 WT.

## Methods

### Cell lines and culture conditions

The human neuroblastoma (NB) cell line SK-N-AS (American Type Culture Collection, Manassas, VA) was maintained in Dulbecco's Modified Eagle Medium (DMEM; Hyclone, Fisher Scientific, Savannah, GA) with 10% fetal bovine serum (Atlanta Biologicals, Lawrenceville, GA) and 2 mM L-glutamine (Hyclone, Fisher Scientific, Savannah, GA) at 37°C and 10% CO_2_. Parent SK-N-AS cells and stable transfectants were cultured under the same conditions. Human dermal microvascular endothelial cells (HDMVEC) were obtained from Lonza, Inc. (Allendale, NJ), and the human bone marrow endothelial cell line, HBMEC-28, was provided by Dr. E. van der Schoot (Sanquin Blood Supply Foundation, The Netherlands) [23]. HDMVEC cells were grown in gelatin-coated culture flasks in Endothelial Cell Grown Medium (EGM) supplemented with 10% heat-inactivated FBS. HBMEC-28 cells were propagated in medium prepared using the EGM™ BulletKit™ (Lonza) according to the directions of the manufacturer.

### Plasmids encoding human ICAM-2 and ICAM-2 variants

The plasmid encoding human ICAM-2 was generated as published [13]. Briefly, cDNA encoding ICAM-2 was generated from RNA isolated from human umbilical vein endothelial cells (Clonetics, San Diego, CA). Primers for full length ICAM-2 were: forward (5'GCTTCCGCTGCCTGGATTCT3') and reverse (5'AAGTCCAGGTGTTGTATTC3'). Amplification was performed at 95°C for 1 min; then 30 cycles of 94°C for 30 sec, 55°C for 30 sec, 72°C for 1 min, followed by 72°C for 7 min. The resulting cDNA was isolated after electrophoresis in agarose gels, sequenced in its entirety by automated sequencing methods (St. Jude Hartwell Center for Bioinformatics, Memphis, TN), and subcloned into the BamH1 restriction site of the expression plasmid pIRESneo2 (Clontech, San Jose, CA) to generate pIRES.ICAM-2. Plasmid transfections were carried out using FuGene6 (Roche Diagnostics, Indianapolis, IN). Forty-eight hours after transfection, 750 μg/ml geneticin (G418; Roche Diagnostics Corporation, Indianapolis, IN) was added to select transfected cell populations. The use of a vector containing an internal ribosomal entry site (IRES) between the ICAM-2 cDNA and the gene encoding the G418 resistance protein eliminated the need to isolate and characterize multiple individual clonal cell lines. The ICAM-2 cDNA sequence was verified as error-free in the resulting cell line (SK-N-ASpIRES.ICAM-2) by the St. Jude Hartwell Center.

### Mutagenesis and nomenclature of ICAM-2 variants

**Figure 2:**
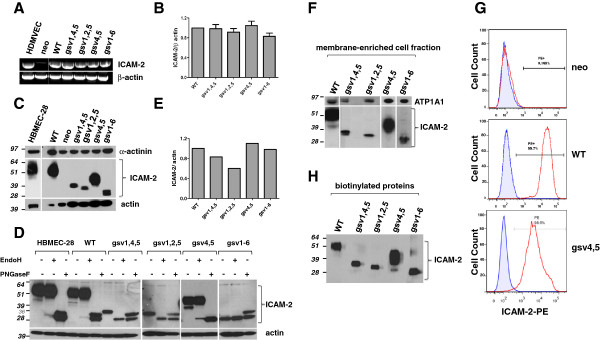
**Transfected SK-N-AS cells express ICAM-2 transcripts and proteins. A**) RNA from control human dermal microvascular endothelial cells (HDMVEC) generated RT-PCR products of the predicted 631 base pairs. All ICAM-2 transfectants contained readily detectable ICAM-2 RNA. **B**) Bar graph depicting quantitation of the RT-PCR products shown in “A”. RNA expression levels were within 20% among the transfectants. This level of variability was not statistically significant. **C**) Immunoblots of whole cell lysates (40 μg protein/lane) demonstrated that control HBMEC-28 cells and WT transfectants expressed immunoreactive protein having an apparent molecular mass of 55-60 kDa. ICAM-2 variants expressed proteins of appropriately lesser apparent masses. Transfectants expressed equivalent levels of actin and α-actinin. **D**) Deglycosylation of proteins in whole cell lysates and subsequent immunoblots for ICAM-2 confirmed that all variants displayed the expected apparent molecular mass of ~30kDa. The “nonspecific” band that appears at ~36kDa in all lanes marked + PNGase F is the PNGase F protein itself. **E**) Quantitation of ICAM-2 variants (lanes indicated as + PNGase F) was done to compare relative amounts of ICAM-2 protein expressed by transfectants. Results were normalized to the level of actin expression for each cell line. **F**) ICAM-2 WT and variants localized to cell membranes. Experimental details are included in Methods. **G**) Membrane localization of ICAM-2 WT and gsv4,5 was confirmed by fluorescence activated cell sorting (FACS) of intact cells, incubated with an antibody recognizing the extracellular domain (ED) of ICAM-2 (CBR-IC2/2) and a PE-conjugated secondary antibody. Non-intact cells were gated out using light scatter parameters and propidium iodide uptake. FACS profiles shown are for PE-positive cells generated using negative control IgG (blue line) or anti-ICAM-2 (red line). **H**) Biotinylation experiments also demonstrated that ICAM-2 WT and variants localized to cell membranes. Experimental details are included in Methods.

**Figure 3:**
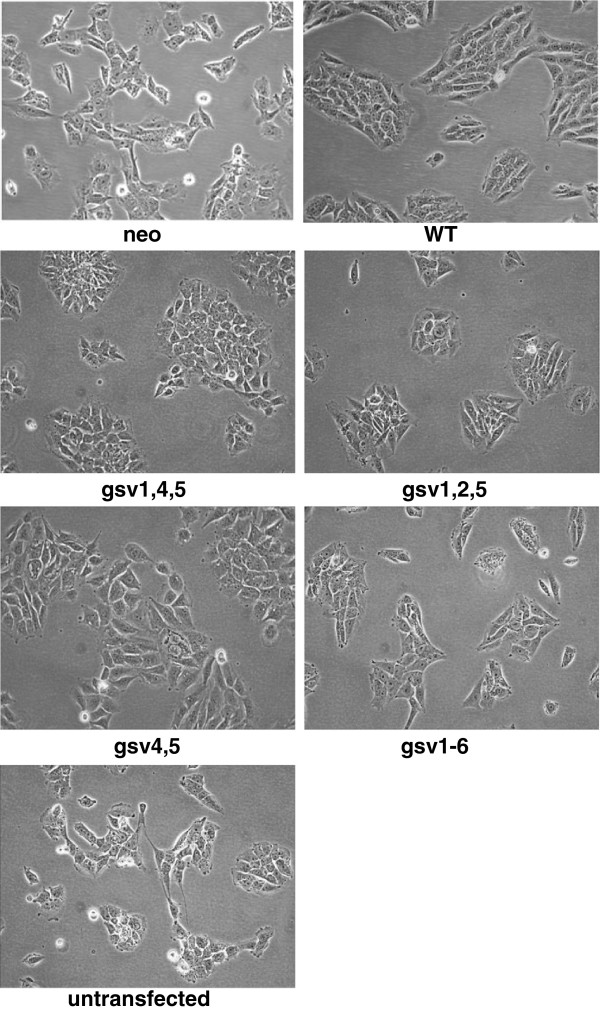
**Morphology of ICAM-2 variants derived from SK-N-AS neuroblastoma cells.** Cells plated at low density (~20% confluence) in CoStar tissue culture flasks were cultured at 37°C at 5% CO_2_ in humidified chambers, and allowed to proliferate to ~70% confluence. SK-N-AS cells transfected to express WT or gsv ICAM-2 have nascent neuronal morphologies. In comparison to SK-N-ASpIRES.ICAM-2 transfectants (WT), gsv transfectants were morphologically more pleomorphic and discohesive. All transfectants grew as attached monolayers. When detached and sized using a Coulter Counter, no differences in cell size were observed.

**Figure 4:**
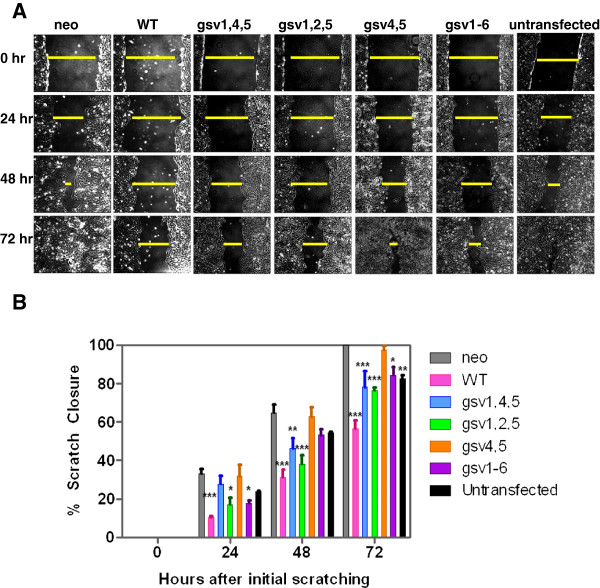
**ICAM-2 WT and glycosylation variants inhibit NB cell migration in scratch assays.** Expression of WT and gsv ICAM-2 inhibited SK-N-AS cell motility *in vitro*. **A**) Scratch assays to evaluate cell motility were performed using standard methods, and images acquired at 24 hour intervals. At each time point, the distance remaining across the scratch was calculated in pixels. Only wells in which initial scratches were of similar width were included in analyses. **B**) Analysis by two-way ANOVA followed by Bonferroni post test showed that at 72 hours post scratch, all variants except gsv4,5 migrated more slowly than transfectants that expressed no detectable ICAM-2 (neo) (P < 0.05* [gsv1-6] and P < 0.001*** [gsv1,4,5 and gsv1,2,5]). At 72 hours post scratch, all gsv transfectants migrated more rapidly than transfectants expressing ICAM-2 WT (P<0.01 – P<0.001, P values not indicated on graph).

**Figure 5:**
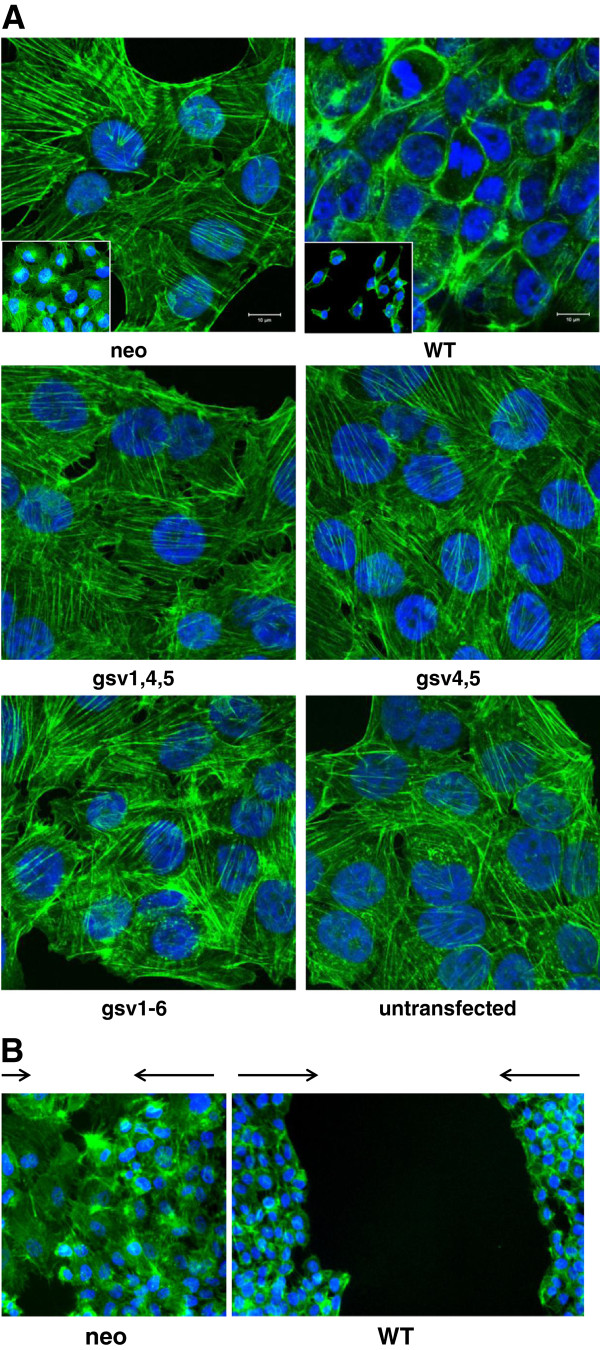
**The cellular distribution of F-actin in control transfectants (neo) differs from that of transfectants expressing ICAM-2 WT. A**) Control neo transfectants harbored transverse actin fibers. ICAM-2 WT induced juxtamembrane actin fiber distribution. ICAM-2 gsv expression did not induce a juxtamembrane localization of actin fibers. Each transfectant was plated at ~60% confluence and maintained under standard conditions at 37^o^C to allow the cells to adhere to chamber slides. Approximately 18 hours after plating, cells were fixed with 3.7% paraformaldehyde and incubated with FITC-conjugated phalloidin. Actin fibers were then visualized using confocal fluorescence microscopy by standard methods. Inset photomicrographs were acquired at 20x magnification and using a Zeiss Axio Observer Z.1 microscope platform in conjunction with Zen 2011 Blue imaging software (Carl Zeiss) (Photomicrographs of control cell lines (neo and WT) were published previously [13]). **B**) The distribution of actin fibers in cells at the leading edge of migration in scratch (wound healing) assays was similar to actin fiber distribution in stationary sub-confluent cultures of each cell line. Scratch assays were performed as described for experiments depicted in Figure 4. At 72 hours “post scratch”, cells were fixed and actin fibers visualized using FITC-phalloidin staining as described above, and fluorescence photomicrographs acquired using Nikon TE2000-U microscope with X-cite 120 mercury vapor short arc in conjunction with Q-capture ProV 5.1.1.14 software (Nikon Instruments, Inc.). Care was taken to acquire images of the control neo transfectants in areas where residual gaps from the original scratch were still visible, to confirm acquisition of the leading edge of migration.

**Figure 6:**
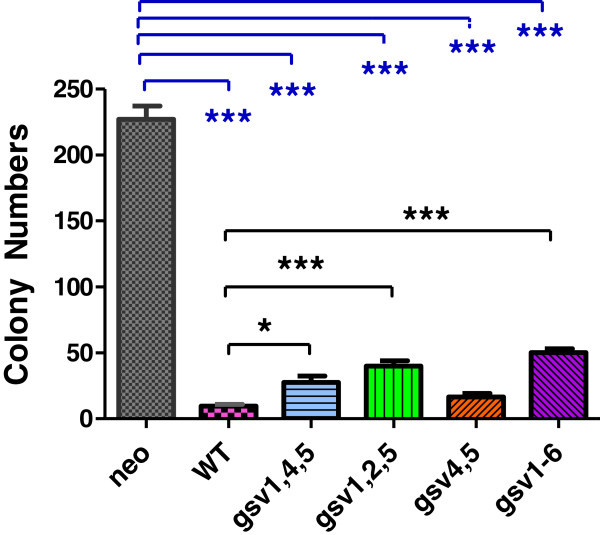
**WT and gsv ICAM-2 suppressed anchorage-independent growth *in vitro*.** All four glycosylation site ICAM-2 variants suppressed anchorage-independent growth. Soft agar assays were performed using standard methods. Number of colonies of >20 cells were visualized 14-21 days after plating and results analyzed using a 2-tailed t test and GraphPad Prism software. P = 0.0124*. P < 0.0001***. Not shown in this Figure are control experiments demonstrating that the growth in soft agar of parental SK-N-AS cells was equivalent to control SK-N-ASpIRESneo transfectants.

**Figure 7:**
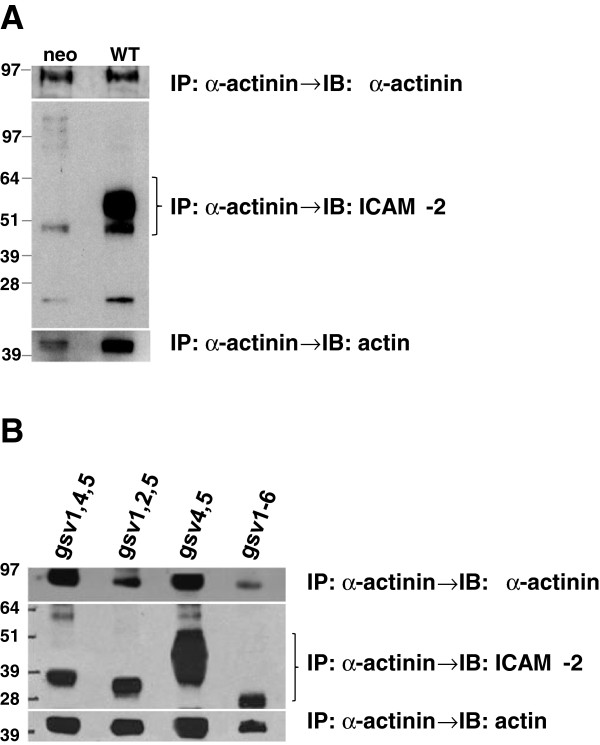
**ICAM-2 WT and variants co-precipitated with α-actinin. A**) IP/IB experiments with control cell lines demonstrate the expected association of ICAM-2, α-actinin and actin in lysates from SK-N-ASpIRES.ICAM-2 cells (labeled as WT), but not SK-N-ASpIRESneo cells (neo). Results for these control cell lines were published previously [13]. **B**) ICAM-2 glycosylation site variants associated simultaneously with α-actinin and actin. Immunoprecipitations (IP) were performed using whole cell lysates and a mouse monoclonal antibody to α-actinin (MAB1682, Millipore). Following protein separation by electrophoresis, immunoblots (IB) were performed with antibodies to α-actinin (sc-7454, Santa Cruz Biotech), ICAM-2 (AF244, R&D Systems), or actin (4968, Cell Signaling). The presence of ICAM-2 WT and variants in each preparation was confirmed by immunoblot analysis of input preparations (a representative blot is shown in Figure 2C) and also by immunoblot analysis of the proteins remaining in the supernatant following precipitation (not shown), to confirm the presence of sufficient/excess ICAM-2 protein or variant in each preparation used for immunoprecipitation experiments.

**Figure 8:**
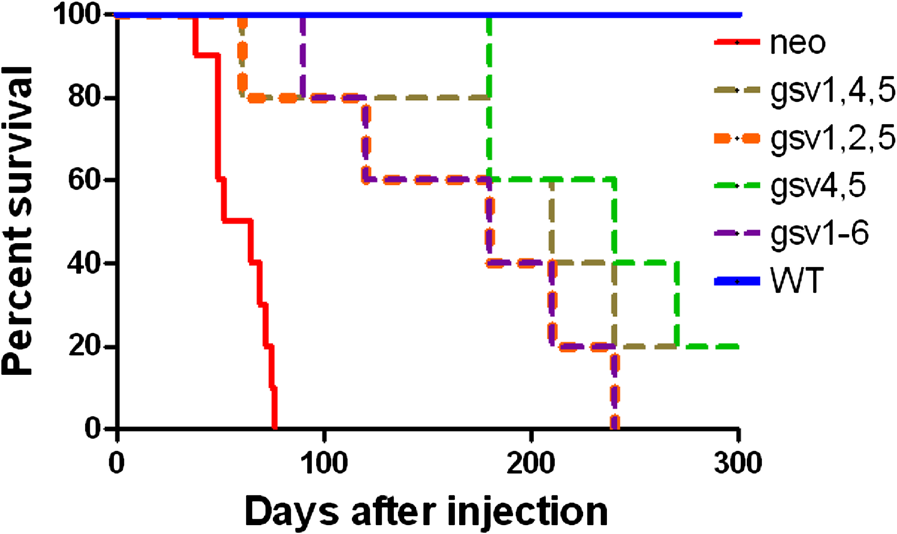
**ICAM-2 variants significantly delayed but did not completely inhibit development of disseminated tumors in an *in vivo *model of metastatic neuroblastoma.** Mice received intravenous injections of SK-N-AS cells expressing no detectable ICAM-2 (neo, N = 10), ICAM-2 WT (N = 10), or one of four hypo-glycosylated forms of ICAM-2 (N = 5/group). Kaplan-Meier survival plots were analyzed by log-rank (Mantel-Cox) test using GraphPad Prism 5 software (Version 5.02). Mice receiving cells expressing hypo-glycosylated ICAM-2 survived longer than mice receiving cells expressing no detectable ICAM-2, but not as long as mice receiving cells expressing ICAM-2 WT.

The objective of this study was to evaluate the impact of the glycosylation status of ICAM-2 on the function in NB tumor cells. Therefore, mutagenesis by overlapping PCR, using Pfu polymerase (QuickChange mutagenesis kit; Stratagene-Agilent Tech, Wilmington, DE) and pIRES.ICAM-2 as a template, was performed to replace some or all of the six asparagine glycosylation sites with alanine residues at positions 47, 82, 105, 153, 176, and 187, each of which is glycosylated in the endogenous wild-type protein. Alanine was chosen for substitution at all six or of a subset of glycosylation sites to prevent glycosylation [24-26]. Plasmids encoding mutated variants of ICAM-2 cDNA were identified by restriction enzyme analysis and sequences confirmed by direct DNA sequencing. Transfection of plasmids encoding ICAM-2 variants into SK-N-AS cells, selection of transfected cells, and culture conditions were the same as for SK-N-ASpIRES.ICAM-2 transfectants. Plasmids and transfected cell lines are designated as glycosylation site variants (gsv) followed by the positions of the amino acids (asparagines) that were replaced with alanine. For example, gsv1,4,5 is non-glycosylated at amino acids 47, 153 and 176 (Figure 1). In Figures 2, 3, 4, 5, 6, 7 and 8, transfected SK-N-AS cell lines expressing variant forms of ICAM-2 are referred to as the specific variant expressed: gsv1,4,5; gsv1,2,5; gsv4,5; and gsv1-6. SK-N-AS cells expressing ICAM-2 WT are labeled in the Figures as “WT”. SK-N-ASpIRESneo2 control transfectants are labeled “neo”.

### Cell proliferation assays (cell doubling time)

To determine if the growth rates of all SK-N-AS transfectants expressing ICAM-2 variants were comparable, 1×10^6^ cells were plated in 75 cm^2^ flasks, and cell numbers determined daily thereafter, using a hemocytometer. Doubling times were calculated using http://www.doubling-time.com/compute.php[27].

### Anchorage-independent growth

Each well of a 6-well plate was plated with 1mL 0.8% Sea-plaque agarose in DMEM with 10% FBS, and cooled to 4°C overnight. NB cells were resuspended in soft agar (0.4%) in DMEM with 10% FBS at a density of 30,000 cells in 10 mL, and 1 mL of cell suspension was overlaid in the wells containing 0.8% agar. Plates were then incubated at 37°C for 14-21 days. Colonies of >20 cells were identified microscopically and the number of colonies/well recorded. Six replicate wells of each transfectant were included in at least three independent experiments, as published previously [13,28].

### Scratch assays (wound healing assays)

To assess the motility of transfectants expressing ICAM-2 variants, we performed scratch or wound healing assays [29]. Cells were plated at an initial density of 1×10^6^ cells per well in 6-well plates and allowed to grow to confluency. A centerline was marked on the underside of each well along its horizontal axis, to designate loci at which images would be acquired at each time point. Vertical linear defects (scratches) were introduced into cell monolayers using a 200 μL pipet tip. Each well received 3 scratches. Images were acquired at 0, 24, 48, and 72 hours post scratch, at 100× magnification at each intersection of the scratch wound (vertical defect) and the marked horizontal centerline using a Dr-5 digital camera (Southern Microscope) mounted t22232o a Nikon Eclipse TS100 inverted microscope. For each time point, 5 measurements were taken per well in each of 3 wells and the average the horizontal width of the linear defect in pixels calculated using Adobe Photoshop CS4 (version 11.0.2).

### Biotinylation of surface proteins

Cells (SK-N-AS cells transfected to express ICAM-2 WT or variants gsv1,4,5; gsv1,2,5; gsv4,5; or gsv1-6) were plated in 175 cm^2^ tissue culture flasks (Corning) with standard growth medium (high glucose DMEM + 10%FBS + 2 mM L-glutamine) and grown to near-confluence. Cells were washed 3 times with ice-cold PBS (pH 8.0) and surface proteins biotinylated for 30 minutes at room temperature using 0.5 mg/ml EZ-Link® Sulfo-NHS-LC-Biotin (21335; Thermo Scientific/Pierce) reconstituted in PBS (pH 8.0). The biotinlyation reaction was quenched by washing the cells 3 times with 100 mM glycine in PBS (pH 7.2). Cells were then lysed with 0.5% NP-40 + complete Mini protease inhibitor (Roche) and homogenized. Biotinylated protein preparations were then incubated with 100 μl Pierce streptavidin® resin (6% crosslinked beaded agarose 50% aqueous slurry) at room temperature for 20 minutes with gentle rocking, and then centrifuged to separate membrane-associated, biotin-avidin precipitated proteins from unlabeled proteins. After unbound proteins (supernatant) were removed, beads were washed 3 times with cold lysis buffer, boiled in sample buffer, and subjected to SDS-PAGE. Following electrophoresis, standard immunoblots were performed.

### Glycosidase digestion

Cleavage of N-linked glycans was carried out using Endo H (#P0702L; New England BioLabs, Ipswich, MA) and PNGase F (#P0704S; New England BioLabs, Ipswich, MA) according to the manufacturer’s instructions. In brief, 20 μg of protein from control cells (HBMEC-28) or NB cells expressing ICAM-2 WT or a variant were denatured at 100°C for 10 minutes with glycoprotein denaturing buffer (5% SDS, 0.4M DTT in PBS). After cooling, protein solutions were centrifuged and incubated for an additional hour at 37°C with 2 μl of either Endo H enzyme (plus G5 Reaction buffer, pH 5.5; New England Biolabs) or PNGase F enzyme (plus G7 reaction buffer, pH 7.5 plus 10% NP-40 ; New England Biolabs). Control samples were subjected to the same denaturing and digesting conditions, but substituting equivalent volumes of water for enzyme. Samples were then subjected to SDS-PAGE and immunoblot analyses.

### Immunoprecipitations and immunoblots

Co-immunoprecipitations (IP) were done as described previously [13]. The antibody used for IP of α-actinin was MAB1682 (Millipore, Fisher Scientific, Savannah, GA). Antibodies used for immunoblots (IB) for ICAM-2, α-actinin, and actin were AF244 (R&D Systems, Minneapolis, MN) and sc-1512 (Santa Cruz Biotech, Santa Cruz, CA), 4968 (Cell Signaling Technology, Danvers, MA), and A5316 (Sigma-Aldrich, St. Louis, MO), respectively. AF244 is a goat polyclonal IgG that recognizes the extracellular domain of ICAM-2. C-20 is a goat polyclonal IgG that recognizes the intracellular C-terminus of the ICAM-2 protein. Secondary HRP-conjugated anti-goat (AF244, sc-1512), anti-rabbit (4968) and anti-mouse (A5316) antibodies were purchased from Santa Cruz Biotech. The ECL system (Pierce ECL Chemiluminescent Substrate; Thermo Scientific) was used to detect presence of horseradish peroxidase (HRP) enzyme. Immunoblot result in Figure 2D was quantitated using Adobe Photoshop CS4 (San Jose, CA) by multiplying area by integrated intensity of the band of interest and normalizing that result to that of a control protein (actin) band.

### Subcellular localization of F-actin

Actin fibers were visualized by fluorescence microscopy using standard methods [30]. Briefly, cells were plated in chamber well slides (2 × 10^5^ cells per chamber) and allowed to adhere. Approximately 18 hours after plating, cells were washed with PBS and fixed with 3.7% (w/v) paraformaldehyde in PBS for 10 minutes at room temperature. Remaining attached cells were washed with PBS, and permeabilized with 0.2% (v/v) Triton X-100 for 1 minute. Actin fibers were stained with 10 μg fluorescein isothiocyanate (FITC)-conjugated phalloidin (P5282; Sigma-Aldrich, St. Louis, MO) in 1 mL phosphate buffered saline (PBS) for 30 minutes. Phalloidin-stained fibers were visualized by confocal fluorescence microscope equipped with narrow band filters, by standard methods (Figure 5A) or by the Zeiss Axio Observer Z.1 microscope platform in conjunction with Zen 2011 Blue imaging software (Carl Zeiss)(insets in Figure 5A). DAPI (4’,6-diamidino-2-phenylindole; appears as blue fluorescence) was used to detect DNA.

### Fluorescence-activated cell sorting (FACS)

Flow cytometric analyses were used to verify the cell membrane localization of ICAM-2 and ICAM-2 variants. Cells (1 × 10^6^) were trypsinized, washed twice with PBS, and incubated for 30 min with goat serum (G9023, Sigma-Aldrich) diluted 1:10 in PBS. Cells were then incubated for 60 minutes on ice with mouse monoclonal anti-human ICAM-2 antibody (CBR-IC2/2, sc-23935 PE; Santa Cruz Biotech). A separate aliquot of cells was incubated with mouse isotope-matched IgG (sc-2025, Santa Cruz Biotech, Santa Cruz, CA) as a negative control. All samples were then washed twice with FACS buffer (1% BSA in PBS) and incubated for 60 minutes on ice with phycoerythrin (PE, sc-3798, Santa Cruz Biotech, Santa Cruz, CA)-conjugated goat anti-mouse IgG. Data were acquired on an LSR-II cytometer (BD Biosciences, San Jose, CA) and analyzed using FlowJo v 7.6.1 (TreeStar) software. Only intact cells, as identified by forward and side light scatter, were analyzed. Propidium iodide (5 μg/mL PBS; stored at 4°C in the dark) staining was also used as a parameter to exclude non-viable cells.

### Morphology

Transfected cells were seeded at a density of 15,000 cells/cm^2^ and maintained under standard culture conditions for 24-48 hours. Photomicrographs were acquired at 20× magnification using a Dr-5 digital camera (Southern Microscope) mounted to a Nikon Eclipse TS100 inverted microscope and archived with TSView software (version 7.1.04, Tucsen Imaging Technology Co./Southern Microscope, Inc., Haw River, NC).

### Membrane fractionation

Cells were washed three times with PBS at 4°C, and resuspended in Cell Lysis Buffer (10 mM HEPES, 10 mM NaCl, 1 mM KH_2_PO_4_, 5 mM NaHCO_3_, 1 mM CaCl_2_, and 0.5 mM MgCl_2_ solution ) + 1 mM EDTA + protease inhibitor (complete mini protease inhibitor tablets; Roche Applied Science, Indianapolis, IN) solution. Cells were allowed to swell for 5 minutes on ice and then homogenized using a Fisher Scientific Misonix XL2000 Ultrasonic Homogenizer (Fisher Scientific), and centrifuged at 7,500 rpm for 5 minutes. The resulting supernatant suspension contained both plasma membrane and cytosolic proteins. This suspension was then re-centrifuged at 25,000 rpm for 30 minutes to separate the plasma membrane (pellet) from the soluble protein fraction. The crude plasma membrane pellet was then resuspended in 40 μl PBS and standard assays done to determine the protein concentration of these suspensions. Samples were stored at -80°C until ready for use. Suspensions were verified as membrane-enriched by immunoblot analysis for the plasma membrane marker (ATP1A1; Novus, Novus Biologicals, Littleton, CO) [31].

### Mouse model of metastatic neuroblastoma

Female *Es1*^*e*^/SCID mice (6-8 weeks old) were obtained from St. Jude Children’s Research Hospital Animal Resources Center (ARC) (Memphis, TN), with the approval of Dr. Phil Potter [32]. The *in vivo* animal studies were carried out in accordance with a protocol approved by the St. Jude Children’s Research Hospital Animal Care and Use Committee (Approval Number: 374). All mice were maintained in the Animal Resources Center at St. Jude and monitored daily for tumor growth. Mice were euthanized as soon as any animal that appeared to be in distress or discomfort. Animals were housed in an AAALAC accredited vivarium at St. Jude Children’s Research Hospital Animal Resources Center under the direction of Dr. Richard Rahija. Bedding and chow were autoclaved and cages changed twice weekly.

When injected intravenously into the tail veins of *Es1*^*e*^/SCID mice, SK-N-AS NB cells (5 × 10^5^ cells) produce disseminated tumors in 100% of mice. The number of cells administered was not toxic to *Es1*^*e*^/SCID mice and no discomfort or death was seen at the time of injection. In this model, there is no “primary tumor” but the pattern of tumor development recapitulates the clinical dissemination of high-risk NB; tumors develop in multiple anatomic locations including lung, liver and bone marrow [33,34]. Groups of 10 (neo and WT transfectants) or 5 mice (gsv1,4,5; gsv1,2,5; gsv4,5; gsv1-6 transfectants) were followed for 300 days after intravenous injection of tumor cells. Any animal that appeared to be in distress was euthanized and the day of euthanasia was recorded as the day of death.

### Statistical analyses

Statistical analyses were performed using GraphPad Prism software, version 5.02 (GraphPad, San Diego, CA); graphs were generated using the same software. Two-tailed t tests were used to analyze results of soft agar assays and two-way ANOVA followed by Bonferroni post tests was used to analyze scratch assays. Soft agar assays and scratch (wound healing) assays were repeated a minimum of three times, with each experiment including six replicates of each cell line. Kaplan Meier survival data were used to calculate the 95% confidence interval for time-dependent fractional survival, using a log-rank (Mantel-Haenszel) test to compare survival of mice receiving vector control cells (neo), ICAM-2 WT, or gsv transfected cells.

## Results and Discussion

### Structural domains of ICAM-2 and ICAM-2 glycosylation site variants (gsv)

The schematic structure of ICAM-2 (Figure 1) reflects the complete 275 amino acid glycoprotein. The indicated domains of this extensively glycosylated protein were discussed in the Background section. The heavy glycosylation status of the ICAM-2 WT protein dictates its migration in SDS-PAGE gels as a diffuse band rather than as a discrete band of specific molecular mass. In this study, as the degree of glycosylation decreases when glycosylation sites are mutated, variants are predicted to migrate at different rates even though the number of amino acids remains unchanged (bottom panel of Figure 1). The non-glycosylated variant gsv1-6 has a predicted molecular mass of 30 kDa.

### ICAM-2 glycosylation site variants (gsv) localize to cell membranes in transfected SK-N-AS neuroblastoma cells

SK-N-AS cells express no detectable endogenous ICAM-2 protein [13]. We transfected these cells to stably express ICAM-2 WT or hypo-glycosylated variants of ICAM-2. RT-PCR analysis of positive control human dermal vascular endothelial cells (HDMVEC) and transfectants expressing ICAM-2 WT or ICAM-2 glycosylation variants (gsv1,4,5, gsv1,2,5, gsv4,5 gsv1-6) demonstrated similar levels of ICAM-2 transcript (Figure 2A, lanes 1,3-7). As expected, neo transfectants (SK-N-AS cells transfected with pIRESneo2 control plasmid) (lane 2) expressed no detectable ICAM-2 RNA. Immunoblot analyses (Figure 2C) done with antibody AF244 (R&D Systems) confirmed protein expression in all cell lines except SK-N-ASpIRESneo. Fully glycosylated endogenous ICAM-2 expressed by HBMEC-28 cells (lane 1) and by transfected ICAM-2 WT cells (lane 2) migrated in SDS-PAGE gels with the expected molecular mass of 55-60 kDa [13]. Immunoblots also demonstrated the expected decrease in apparent molecular mass of hypo-glycosylated ICAM-2 variants compared to full length ICAM-2. As the extent of glycosylation decreased, immunoreactive bands appeared less diffuse and of lower mass. The non-glycosylated variant expressed by gsv1-6 cells migrated at ~30 kDa, as expected for a protein of 254 amino acids (275 amino acid nascent protein – the 21 amino acid signal peptide). Notably, differences in level of glycosylation preclude direct comparison of levels of protein expression among the transfectants. Furthermore, the unexpected area/intensity of the immunoreactive band in the lane containing lysate from gsv4,5 cells suggest the possibility of N-glycan heterogeneity among the differentially glycosylated ICAM-2 variants. Immunoblots for actin and α-actinin served as loading controls.

Next, we examined ICAM-2 N-glycan sensitivity to endoglycosidase H (Endo H) and N-glycosidase F (PNGase F) since modification of glycosylation status can be detected using these enzymes. Endo H hydrolyzes high mannose and hybrid N-glycans synthesized in the endoplasmic reticulum (ER), while PNGase F hydrolyzes all N-linked glycans including complex glycans synthesized in the Golgi. ICAM-2 expressed by all transfectants and HBMEC-28 cells were sensitive to PNGase F and displayed increased electrophoretic mobility following a 1 hour incubation with PNGase F (Figure 2D), with non-glycosylated forms of ICAM-2 migrating at ~30 kDa. (The “nonspecific” band that appears at ~36kDa in all lanes marked + PNGase F is the PNGase F protein itself.) In contrast to results with PNGase F, results following incubation of cell lysates with Endo H differed for forms of ICAM-2 that were glycosylated at position 47 compared with those that were not glycosylated at this position. ICAM-2 WT and gsv4,5 and ICAM-2 from control HBMEC-28 cells were Endo H resistant, while gsv1,4,5 and gsv1,2,5 were Endo H sensitive. Potentially, N-glycans of ICAM-2 modify protein conformation thereby impacting protein stability, in addition to their documented roles in ligand recognition. Comparison of results from gsv4,5 with gsv1,2,5 and gsv1,4,5 suggests that N-glycans at amino acid 47 may be the most critical with respect to protein conformation and stability. Interestingly, quantitation of this experiment (Figure 2E) by densitometric scanning demonstrated that the level of gsv1,2,5 expressed was ~60% that of WT protein and the level of gsv1,4,5 was ~83% that of WT protein. Possibly this observation reflects a decreased protein stability of these variants, each of which contained an amino acid substitution at position 47 that prevented glycosylation at this site. Further, the robust level of expression of variant gsv4,5, the only variant that did not contain an amino acid substitution at position 47, is consistent with the hypothesis that N-glycosylation at this position may be critical to ICAM-2 protein structure and stability. Our data comparing Endo H and PNGase F sensitivities of ICAM-2 WT and variants suggest that N-glycosylation site at amino acid 47 may be the most critical N-glycan structure with respect to protein stability.

Next, we showed that similar to the WT protein, hypo-glycosylated ICAM-2 variants were associated with the plasma membrane (Figure 2F, 2G, 2H). We first isolated membrane-enriched fractions of each transfectant and performed immunoblots for ICAM-2 and also for the plasma membrane marker ATP1A1 (Na+/K+ transporting ATPase subunit alpha 1; Novus Biologicals, Littleton, CO). Membrane-enriched fractions contained readily detectable levels of ATP1A1 and also ICAM-2 proteins of the expected molecular mass using an antibody that recognizes the CD domain of ICAM-2 (sc-1512; Santa Cruz Biotech) (Figure 2F). To corroborate these data, we performed immunofluorescence staining using intact transfected cell lines and an antibody that recognized the ED of ICAM-2 (CBR-IC2/2-PE). Flow cytometric analysis of WT, neo and each of the four gsv transfectants demonstrated measurable levels of antibody reactivity (cell surface expression in intact cells) for ICAM-2 protein only for cells expressing WT and gsv4,5 (Figure 2G), even though gsv1,4,5, gsv1,2,5, and gsv1-6 were clearly seen by immunoblot. The CBR-IC2/2 antibody was generated using full length ICAM-2 (Santa Cruz Biotech). This antibody recognizes the LFA-1 binding domain of ICAM-2 which includes amino acid residues #42-63 within extracellular IgLD-1 domain [17,35-38], and inhibits binding of LFA-1 to ICAM-2. Our FACS results show that the CBR-IC2/2 did not react with any of the three variants containing an amino acid substitution at position 47 (gsv1,4,5, gsv1,2,5 and gsv1-6), suggesting that substitution at this position destroyed antibody recognition. However, since gsv4,5 contained the endogneous Asn at position 47, we used CBR-IC2/2 to confirm membrane localization of the expressed variant. The data are consistent with the previous report [17,35-38] that antibody CBR-IC2/2 recognizes amino acid residues within IgLD-1 and that an Asn residue at position 47 is essential for that recognition.

Next, cell surface protein biotinylation was performed to confirm cell membrane surface localization of all ICAM-2 variants. As shown in Figure 2H, biotinylation of proteins of intact cells showed readily detectable ICAM-2 proteins with the expected electrophoretic mobility for all cell lines. Data in Figures 2F, 2G, and 2H indicate that ICAM WT and variants localize to cell membranes.

Taken together, the data indicate that variable glycosylation does not affect the subcellular localization of ICAM-2 and that variants localize to the plasma membrane similar to the endogenous protein. As expected, control-transfected SK-N-AS cells showed no detectable ICAM-2 expression.

### Morphology and cell doubling time of transfected gsv cell lines

Having confirmed expression of transfected cDNAs and membrane localization of encoded proteins, we further characterized transfectants with respect to cell morphology and cell doubling time. Photomicrographs (Figure 3) demonstrate that all six transfected SK-N-AS cell lines and the untransfected SK-N-AS parent cell line grow as attached monolayers. In general, all seven also showed similar nascent neuronal morphology, were similar in size when detached, and had doubling times of 35.0 ± 4.4 hours. The single exception to this was the gsv1,2,5 cell line, which had a doubling time of 59 hours. Interestingly, in spite of being similar in size when detached, the images in Figure 3 suggest that gsv1,4,5 and gsv1,2,5 transfectants were more adhesive than the ICAM-2 variant transfectants, and appeared smaller in size when attached and grew as aggregates more similar to WT transfectants rather than the more diffuse monolayers of untransfected and control neo transfectants. In contrast, gsv1-6 transfectants appeared morphologically more similar to neo controls than to WT transfectants.

Of particular interest, SK-N-ASpIRESneo cells and gsv transfectants appeared more discohesive and pleomorphic than cells expressing ICAM-2 WT. Also, SK-N-ASpIRESneo cells and gsv transfectants had relatively non-smooth borders compared to cells expressing ICAM-2 WT. Cells expressing gsv4,5 demonstrated a “drunken honey comb” appearance, a description frequently appropriate to malignant cells with decreased cohesiveness and non-smooth borders. Cells expressing gsv1-6, grew in smaller clusters than other transfectants. The most consistent findings were the relatively discohesive and pleomorphic characteristics of SK-N-AS cells expressing gsv transfectants, compared to ICAM-2 WT, possibly indicative of a more motile phenotype than that of cells expressing the WT protein.

To address this possibility and to more completely characterize the functional phenotype of the gsv transfectants, we characterized each transfectant using assays that reflect specific aspects or cellular functions involved in metastasis. These assays included cell motility (wound healing or scratch assays), anchorage-independent growth in soft agar, subcellular localization of actin fibers (F-actin), and development of disseminated tumors in an *in vivo* model of metastatic NB.

### ICAM-2 variants altered tumor cell motility but did not affect actin fiber distribution *in vitro*

Cell motility is a primary determinant of metastatic potential [39-41]. The actin cytoskeleton, in turn, is a primary determinant of cell motility. Therefore, we compared the relative motility of the parental cell line and all six transfectants in scratch (wound healing) assays (Figures 4A, 4B). We also assessed the subcellular distribution of F-actin by staining actin fibers with FITC-conjugated phalloidin (Figure 5A) and the actin fiber distribution of cells at the leading edge of migration in wound healing assays (Figure 5B) to evaluate potential differences between neo and WT transfectants.

First, in standard scratch assays the width of the scratched area that remained free of cells at 24 hour intervals after introducing the scratch was quantitated in pixels. Results were expressed as the degree to which the scratch had closed, at 24 hour intervals post scratch (% scratch closure). SK-N-ASpIRESneo cells which express no detectable ICAM-2 completely closed the scratch within 72 hours (Figure 4A). In contrast, SK-N-ASpIRES.ICAM-2 cells migrated more slowly (P < 0.001, compared to SK-N-ASpIRESneo cells), with only 56% of the initial scratch filled at 72 hours (Figure 4B). In general, cells expressing the gsv ICAM-2 constructs migrated more slowly than control SK-N-ASpIRESneo cells and more rapidly than SK-N-ASpIRES.ICAM-2 (WT) cells. P values ranged from < 0.05-0.001 when cell lines expressing gsv constructs were compared with either control cell line. The single exception was the lack of a significant difference in the motility of cells expressing gsv4,5 and cells expressing no detectable ICAM-2 (neo, in Figure 4) at any time point. Simple differences in cell doubling times do not account for the apparent lack of function (failure to inhibit migration) of gsv4,5, as the doubling time for these cells did not differ from other transfectants.

gsv4,5 was the only construct evaluated that did not contain an Asn → Ala substitution at amino acid 47, suggesting that glycosylation at this site minimizes or circumvents the ability of ICAM-2 to inhibit NB cell motility. Possibly, glycosylation at position 47 inhibits adhesion, but this effect may be modulated by glycosylation at other sites in the WT protein. However, since ICAM-2 WT is glycosylated at this site and the WT protein significantly inhibits NB cell motility, the data are not consistent with a straightforward correlation between glycosylation of this specific residue and cell motility, as reflected by this assay. The data demonstrate that NB cell motility was most affected by fully glycosylated ICAM-2, but that three of the hypo-glycosylated ICAM-2 constructs retained an attenuated function compared to the endogenous fully-glycosylated protein.

Second, we performed phalloidin staining assays to determine the subcellular distribution of actin fibers. The ICAM-2-mediated decrease in NB cell motility suggested a possible involvement of the actin cytoskeletal network, a primary regulator of cell motility; and our previous work demonstrated a correlation between transverse distribution of actin fibers and a relatively motile metastatic phenotype in a panel of NB cell transfectants. As a preliminary assessment of actin cytoskeletal function, we examined the subcellular localization of actin fibers in cells expressing ICAM-2 variants. Fluorescence photomicrographs of F-actin in control cell lines (neo and WT cells in Figure 5) [13] stained with FITC-conjugated phalloidin demonstrated the expected contrast in distribution of actin fibers; relatively motile SK-N-ASpIRESneo cells harbored transverse actin fibers, while relatively non-motile SK-N-ASpIRES.ICAM-2 cells had juxtamembrane actin fibers. If the scratch assay and F-actin distribution accurately reflect cell motility, then scratch assay results would predict that cells expressing gsv4,5 would have transverse actin fibers and the other three constructs would have juxtamembrane fibers. FITC-conjugated phalloidin staining clearly demonstrated that this prediction did not hold true, and that cells expressing all four ICAM-2 variants had transverse actin fiber distribution (Figure 5 and data not shown). This assay does not provide a simple preliminary screen to assess the impact of ICAM-2 WT or gsv on NB cell motility *in vitro*.

We then also addressed whether the observed differences in actin fiber distribution could be attributed to differences in cell density. We plated neo transfectants at twice the original density and ICAM-2 cells at half the original density shown in the insets of the two upper panels in Figure 5A. Altering cell density did not affect actin fiber distribution. Actin fiber distribution appeared to be unrelated to cell density. As an alternative approach to examine the effect of cell density on F-actin distribution in the neo and ICAM-2 WT transfectancts we again performed a scratch assay as shown in Figure 4, but just prior to complete closure of the scratch by neo transfectants (~72 hours post scratch), we fixed both neo and ICAM-2 WT transfectants and incubated the cells with FITC-conjugated phalloidin. The question to be addressed was whether the F-actin distribution of cells at the leading edge of migration would be similar to its distribution in non-migrating cells. Fluorescence photomicrographs in Figure 5B show that both non-migrating and migrating SK-N-ASpIRESneo cells display transverse actin fibers and both non-migrating and migrating SK-N-ASpIRES.ICAM-2 cells display juxtamembrane actin fibers.

### All four ICAM-2 variants inhibited colony growth in anchorage-independent growth assays

We next assessed anchorage-independent colony growth *in vitro*, an assay that often reflects tumorigenic potential *in vivo* in preclinical models. Of note, while somewhat controversial, multiple published studies have also demonstrated correlations between growth in soft agar and metastatic, rather than tumorigenic potential [42-44]. Our previously published data demonstrate that ICAM-2 transfectants with decreased metastatic potential *in vivo* also produce fewer colonies in soft agar *in vitro*[13]. A plausible explanation consistent with observed correlations between anchorage-independent growth *in vitro* and metastatic potential *in vivo* is that relatively motile cells show decreased sensitivity to loss of anchorage dependency and therefore a greater capacity to initiate colony growth. The aggregation of sparsely plated cells precedes colony growth, and cells that more readily aggregate (relatively motile cells) produce more viable colonies in soft agar. Therefore, it was of interest to determine whether hypo-glycosylated ICAM-2 variants suppressed growth in soft agar to the same extent as ICAM-2 WT. As expected, cells expressing fully-glycosylated ICAM-2 WT formed fewer colonies in soft agar (9.6 ± 1.4) than control transfectants (227 ± 10.1 colonies) (P< 0.0001***, Figure 6). The number of colonies produced by all variants was intermediate to the number of colonies produced by cells expressing the WT and cells expressing no detectable ICAM-2. Hypo- and non-glycosylated variants produced significantly fewer colonies than control transfectants (P < 0.0001), but significantly more colonies than SK-N-ASpIRES.ICAM-2 cells (P < 0.05 - < 0.0001). The single exception to this observation was that cells expressing gsv4,5 produced the similar number of colonies (16.7 ± 2.6) as SK-N-ASpIRES.ICAM-2 cells. Hypo-glycosylated ICAM-2 suppressed anchorage-independent growth, but the degree of suppression was less than that induced by ICAM-2 WT. We concluded that all forms of ICAM-2 were functional in this assay, and that the cells expressing each of the four variants needed to be characterized using an *in vivo* model of metastatic NB.

### ICAM-2 WT and all four variants associate with α-actinin and actin

Prior to undertaking these *in vivo* experiments, we addressed a more mechanistic aspect of ICAM-2 function in NB cells. Our previous work demonstrated that the α-actinin binding site in the cytoplasmic domain (CD) of ICAM-2 is essential to ICAM-2-mediated suppression of disseminated tumor development in an *in vivo* model of metastatic NB [13]. When the α-actinin binding site of ICAM-2 is intact, ICAM-2 associates with α-actinin and actin simultaneously and suppresses the metastatic properties of NB cells. Having observed that the four gsv transfectants retained the function of the WT protein in either scratch or anchorage-independent growth assays or both, we postulated that the glycosylation status of ICAM-2 would not affect its association with α-actinin and actin in NB cells. To address this hypothesis, we performed immunoprecipitations (IP) with an antibody to α-actinin, and then immunoblots (IB) to determine whether gsv ICAM-2 variants associated with α-actinin and actin. IP/IB results for positive (WT) and negative (neo) control cell lines (Figure 7A) demonstrate the anticipated interaction of ICAM-2, α-actinin and actin compared to minimal interaction of α-actinin and actin when ICAM-2 was not expressed. IP/IB results (Figure 7B) showed that all ICAM-2 variants associated with α-actinin and actin.

In interpreting the IP/IB data, we note that the data are qualitative and do not necessarily reflect the ratio in which these proteins associate for several reasons. Firstly, immunoblots for glycosylated ICAM-2 give an area x density signal approximately 300% - 400% greater than that for the same amount of de-glycosylated ICAM-2 (Figure 2C). Secondly, α-actinin induces actin self-association [45], resulting in variable numbers of actin molecules associated with a single α-actinin molecule. Thirdly, the necessary use of mono- and polyclonal antibodies likely allows binding of secondary antibodies to a variable number of recognition sites on primary antibodies. However, the data do clearly indicate that all four variants associate with α-actinin, an interaction essential to ICAM-2-mediated suppression of metastatic potential. Based on this mechanistic consideration and on cell function assays (Figures 4 and 6), we would predict that all hypo-glycosylated ICAM-2 variants retain at least partial function, in suppressing metastatic properties of NB cells. Next, we tested this hypothesis in a murine model of metastatic NB. Of note, based on co-IP data in Figure 7, ICAM-2 WT and gsv4,5 appear to interact more readily with α-actinin than do the other three hypo-glycosylated ICAM-2 variants. We next evaluated the function of ICAM-2 WT and variants in an *in vivo* model of metastatic neuroblastoma.

### Hypo- and non-glycosylated ICAM-2 delay tumor progression in a preclinical model of metastatic neuroblastoma

To determine the extent to which N-linked glycosylation impacts the metastatic phenotype of ICAM-2 expressing NB cells *in vivo*, we injected each of the SK-N-AS NB cell transfectants intravenously into 6-8 week old *Es1*^*e*^/SCID mice, and observed the mice for tumor development. As expected, Kaplan-Meier survival plots demonstrated that mice receiving control cells (N = 10) that express no detectable ICAM-2 (Figure 8, neo [solid red line]) developed tumors at multiple anatomic sites and required euthanasia within 2-3 months. Also as expected, mice receiving cells that express ICAM-2 WT (N = 10) developed no tumors and survived significantly longer (300 days, P < 0.001 compared to controls). Interestingly, expression of hypo-glycosylated ICAM-2 delayed, but did not prevent, development of disseminated tumors in this *in vivo* model. More specifically, when mice were injected with control neo transfectants, tumors became evident by non-invasive imaging within 30-60 days (data not shown). Euthanasia of mice in this group was required as early as day 38. In contrast, mice injected with gsv4,5 transfectants did not develop detectable tumors or require euthanasia until day ~200. At day 300, the termination date of the experiment, no tumor was evident in a subset of this group of mice. The similarity in function of WT and gsv4,5 in anchorage-independent growth assays (Figure 6), interaction with α-actinin (Figure 7), and the *in vivo* survival assay (Figure 8) suggest the hypothesis that glycosylation of ICAM-2 at amino acid 47 contributes significantly to the function of ICAM-2 in NB cells. However, overall, mice receiving cells expressing each of the glycosylation site variants survived longer than control cells (P ≤ 0.0002), but not as long as mice receiving cells expressing ICAM-2 WT (P ≤ 0.05). Hypo-glycosylation attenuated but did not abolish the ability of ICAM-2 to suppress metastatic properties of NB cells.

## Conclusions

While *in vitro* cell motility (scratch assay) most accurately predicted cell phenotype *in vivo*, no single *in vitro* assay consistently predicted results *in vivo* for all ICAM-2 variants. In general, the morphology, anchorage-independent growth, and motility of cells expressing hypo- or non-glycosylated variants of ICAM-2 were intermediate to characteristics of negative and positive control transfectants expressing no detectable ICAM-2 or the WT protein. *In vivo* results for all transfectants were also intermediate to these control cell lines, in that expression of gsv transfectants significantly delayed but did not prevent the occurrence of disseminated disease in a model of metastatic NB. In addition, our results from Endo H sensitivity studies suggest that glycosylation at position 47 may be critical to protein folding or conformation, and that glycosylation at this position likely affects protein stability. Potentially, a decrease in ICAM-2 protein stability leads to lower levels of membrane-associated ICAM-2, which, in turn, impacts the novel role of ICAM-2 in suppressing metastatic properties. We conclude that the glycosylation status of ICAM-2 significantly affects the function of this protein in SK-N-AS NB cells.

## Abbreviations

ICAM-2: Intercellular adhesion molecule-2, CD102; NB: Neuroblastoma; ED: Extracellular domain; Sp: Signal peptide; TD: Transmembrane domain; CD: Cytoplasmic domain; CAM: Cell adhesion molecules; Gsv: Glycosylation site variant; IgLD-1 and IgLD-2: Two immunoglobulin-like C2 domains; IB: Immunoblot; IP: Immunoprecipitation; Asn N: Asparagine; Ala A: Alanine.

## Competing interests

The authors declare that they have no competing interests.

## Authors’ contributions

JMF, PLG, SBB, SB and KJY conducted the study. JMF, LNC and KJY analyzed the data. KJY designed the study and wrote the manuscript. All authors read and approved the final manuscript.

## Pre-publication history

The pre-publication history for this paper can be accessed here:

http://www.biomedcentral.com/1471-2407/13/261/prepub

## References

[B1] Kawauchi T Cell adhesion and its endocytic regulation in cell migration during neural development and cancer metastasis Int J Mol Sci 2012 13 4 4564 4590 22605996 10.3390/ijms13044564 PMC3344232

[B2] Dallas MR Liu G Chen WC Thomas SN Wirtz D Huso DL Konstantopoulos K Divergent roles of CD44 and carcinoembryonic antigen in colon cancer metastasis FASEB J 2012 26 6 2648 2656 10.1096/fj.12-203786 22415308 PMC3360151

[B3] Leda J Yokoyama S Tamura K Takifuji K Hotta T Matsuda K Oku Y Nasu T Kiriyama S Yamamoto N Nakamura Y Shively JE Yamaue H Re-expression of CEACAM1 long cytoplasmic domain isoform is associated with invasion and migration of colorectal cancer Int J Cancer 2011 129 6 1351 1361 10.1002/ijc.26072 21413011

[B4] Ngora H Galli UM Miyazaki K Zoller M Membrane-bound and exosomal metastasis-associated C4.4A promotes migration by associating with the α(6)β(4) integrin and MT1-MMP Neoplasia 2012 14 2 95 107 22431918 10.1593/neo.111450 PMC3306255

[B5] Dalva MB McClelland AC Kayser MS Cell adhesion molecules: signaling functions at the synapse Nature Rev Neuroasience 2007 8 206 220 10.1038/nrn2075 PMC4756920 17299456

[B6] Williams AF Barclay AN The immunoglobulin superfamily-domains for cell surface recognition Ann Rev Immunol 1988 6 381 405 10.1146/annurev.iy.06.040188.002121 3289571

[B7] Casasnovas JM Springer TA Liu J Harrison SC Wang J Crystal structure of ICAM-2 reveals a distinctive integrin recognition surface Nature 1997 387 15 312 315 9153399 10.1038/387312a0

[B8] Nortamo P Salcedo R Timonen T Patarroyo M Gahmberg CG A monoclonal antibody to the human leukocyte adhesion molecule intercellular adhesion molecule-2 J Immunol 1991 146 2530 2535 2016518

[B9] de Fougerolles AR Stacker SA Schwarting R Springer TA Characterization of ICAM-2 and evidence for a third counter-receptor for LFA-1 J Exp Med 1991 174 253 257 10.1084/jem.174.1.253 1676048 PMC2118873

[B10] Kotovuori A Pessa-Morikawa T Kotovuori P Nortamo P Gahmberg CG ICAM-2 and a peptide from its binding domain are efficient activators of leukocyte adhesion and integrin affinity J Immunol 1999 162 11 6613 6620 10352278

[B11] Reiss Y Engelhardt B T cell interaction with ICAM-1-deficient endothelium in vitro: transendothelial migration of endothelial ICAM-1 and ICAM-2 Int Immunol 1999 11 9 1527 1539 10.1093/intimm/11.9.1527 10464174

[B12] Huang M-T Larbi KY Scheiermann C Woodfin A Gerwin N ICAM-2 mediates neutrophils transmigration in vivo: evidence for stimulus specificity and a role in PECAM-2-independent transmigration Blood 2006 107 4721 4727 10.1182/blood-2005-11-4683 16469869

[B13] Yoon KJ Phelps DA Bush RA Remack JS Billups CA Khoury JD ICAM-2 expression mediates a membrane-actin link, confers a nonmetastatic phenotype and reflects favorable tumor stage or histology in neuroblastoma PLoS One 2008 3 11 e3629 10.1371/journal.pone.0003629 18978946 PMC2575377

[B14] Huang MT Mason JC Birdsey GM Amsellem V Gerwin N Haskard DO Ridley AJ Randi AM Endothelial intercellular adhesion molecule (ICAM)-2 regulates angiogenesis Blood 2005 106 5 1636 1643 10.1182/blood-2004-12-4716 15920013

[B15] Yonemura S Hirao M Doi Y Takahashi N Kondo T Ezrin/radixin/moesin (ERM) proteins bind to a positively charged amino acid cluster in the juxta-membrane cytoplasmic domain of CD44, CD43, and ICAM-2 J Cell Biol 1998 40 885 895 9472040 10.1083/jcb.140.4.885 PMC2141743

[B16] Heiska L Kantor C Parr T Critchley DR Vilja P Binding of the cytoplasmic domain of intercellular adhesion molecule-2 (ICAM-2) to a-actinin J Biol Chem 1999 271 26214 26219 8824270 10.1074/jbc.271.42.26214

[B17] Jimenez D Roda-Navarro P Springer TA Casasnovas JM Contribution of N-linked glycans to the conformation and function of intercellular adhesion molecules (ICAMs) J Biol Chem 2005 280 7 5854 5861 15545280 10.1074/jbc.M412104200

[B18] Jamal BT Nita-Lazar M Gao Z Amin B Walker J Kukuruzinska MA N-glycosylation status of E-cadherin controls cytoskeletal dynamics through the organization of distinct β-catenin-and γ-catenin-containing AJs Cell Health Cytoskelet 2009 1 67 80 20502620 10.2147/chc.s5965 PMC2874908

[B19] Zhao H Liang Y Xu Z Wang L Zhou F Li Z Jin J Yang Y Fang Z Hu Y Zhang L Su J Zha X N-glycosylation affects the adhesive function of E-cadherin through modifying the composition of adherens junctions (AJs) in human breast carcinoma cell line MDA-MB-435 J Cell Biochem 2008 104 162 175 10.1002/jcb.21608 17979184

[B20] Horst AK Bickert T Brewig N van Rooijen N Schumacher U Beauchemin N Ito WD Fleischer B Wagner C Ritter U CEACAM1+ myeloid cells control angiogenesis in inflammation Blood 2009 113 26 6726 6736 10.1182/blood-2008-10-184556 19273835

[B21] Guo H-B Lee I Kamar M Akiyama SK Pierce M Aberrant N-glycosylation of b1 integrin causes reduced a5b1 integrin clustering and stimulate cell migration Cancer Res 2002 62 6837 6845 12460896

[B22] Nita-Lazar M Noonan V Rebustini I Walker J Menko AS Kukuruzinska MA Overexpression of DPAGT1 leads to aberrant N-glycosylation of E-cadherin and cellular discohesion in oral cancer Cancer Res 2009 69 14 5673 5680 10.1158/0008-5472.CAN-08-4512 19549906 PMC2771190

[B23] Rood PM Calafat J von dem Borne AE Gerritsen WR van der Schoot CE Immortalisation of human bone marrow endothelial cells: characterization of new cell lines Eur J Clin Invest 2000 30 618 629 10.1046/j.1365-2362.2000.00672.x 10886302

[B24] Polanska UM Duchesne L Harries JC Fernig DG Kinnunen TK N-glycosylation regulates fibroblast growth factor receptor/EGL-15 activity in Caenorhabditis elegans in vivo J Biol Chem 2009 284 48 33030 33039 10.1074/jbc.M109.058925 19801543 PMC2785143

[B25] Skropeta D Settasatian C McMahon MR Shearston K Caiazza D McGrath KC Jin W Rader DJ Barter PJ Rye KA N-glycosylation regulates endothelial lipase-mediated phospholipid hydrolysis in apoE- and apoA-I-containing high density pipoproteins J Lipid Res 2007 48 2047 2057 10.1194/jlr.M700248-JLR200 17545692

[B26] Morrison KL Weiss GA Combinational alanine-scanning Curr Opin Chem Biol 2001 5 302 307 10.1016/S1367-5931(00)00206-4 11479122

[B27] Mohamet L Lea ML Ward CM Abrogation of E-cadherin-mediated cellular aggregation allows proliferation of pluripotent mouse embryonic stem cells in shake flask bioreactors PLoS One 2010 5 9 e12921 10.1371/journal.pone.0012921 20886069 PMC2944850

[B28] Hong X Jiang F Kalkanis SN Zhang ZG Zhang X Zheng X Jiang H Mikkelsen T Chopp M Increased chemotactic migration and growth in heparanase-overexpressing human U251n glioma cells J Exp Clin Cancer Res 2008 27 1 23 10.1186/1756-9966-27-23 18647407 PMC2499998

[B29] Liang CC Park AY Guan JL In vitro scratch assay: a convenient and inexpensive method for analysis of cell migration in vitro Nat Protoc 2007 2 2 329 333 10.1038/nprot.2007.30 17406593

[B30] Moralli D Monaco ZL Simultaneous detection of FISH signals and bromo-depxyuridine incorporation in fixed tissue cultured cells PLoS One 2009 4 2 e4483 10.1371/journal.pone.0004483 19221585 PMC2637417

[B31] Heineke J Auger-Messier M Correll RN Xu J Benard MJ Yuan W Drexler H Parise LV Molkentin JD CIB1 is a regulator of pathological cardiac hypertrophy Nat Med 2010 16 8 872 879 10.1038/nm.2181 20639889 PMC2917617

[B32] Morton CL Lacono L Hyatt JL Taylor KR Cheshire PJ Houghton PJ Danks MK Stewart CF Potter PM Activation and antitumor activity of CPT-11 in plasma esterase-deficient mice Cancer Chemother Pharmacol 2005 56 629 636 10.1007/s00280-005-1027-y 15918039

[B33] Wagner LM Guichard SM Burger RA Morton CL Straign CM Ashmun RA Harris LC Houghton PJ Potter PJ Danks MK Efficacy and toxicity of a virus-directed enzyme prodrug therapy purging method: preclinical assessment and application to bone marrow samples from neuroblastoma patients Cancer Res 2002 62 5001 5007 12208753

[B34] Dickson PV Hammer JB Burger RA Garcia E Ouma AA Kim SU Ng CYC Gray JT Aboody KS Danks MK Davidoff AM Intravascular administration of tumor tropic neural progenitor cells permits targeted delivery of interferon-β and restricts tumor growth in a murine model of disseminated neuroblastoma J Ped Surgery 2007 42 48 53 10.1016/j.jpedsurg.2006.09.050 17208540

[B35] Li R Nortamo P Valmu L Tolvanen M Huuskonen J Kantor C Gahmberg CG A peptide from ICAM-2 binds to the leukocyte integrin CD11a/CD18 and inhibits endothelial cell adhesion J Biol Chem 1993 268 17513 17518 8349630

[B36] Diacovo AG de Fougerlles AR Bainton DF Springer TA A functional integrin ligand on the surface of platelets: Intercellular adhesion molecule-2 J Clin Invest 1994 94 1243 1251 10.1172/JCI117442 8083366 PMC295209

[B37] Casasnovas JM Piersono C Springer TA Lymphatic function-associated antigen-1 binding residues in intercellular adhesion molecule-2 (ICAM-2) and the integrin binding surface in the ICAM family Proc Natl Acad Sci USA 1999 96 3017 3022 10.1073/pnas.96.6.3017 10077629 PMC15887

[B38] Bocci G Nicolaou KC Kerbel RS Protracted low-dose effects on human endothelial cell proliferation and survival in vitro reveal a selective antiangiogenic window for various chemotherapeutic drugs Cancer Res 2002 62 23 6938 6943 12460910

[B39] Ridley AJ Schwartz MA Burridge K Firtel RA Ginsberg MH Borisy G Parsons JT Horwitz AR Cell migration: Integrating signals from front to back Science 2003 302 1704 1709 10.1126/science.1092053 14657486

[B40] Friedl P Wolf K Plasticity of cell migration: a multiscale tuning model J Cell Biol 2009 188 1 11 19 19951899 10.1083/jcb.200909003 PMC2812848

[B41] Stevenson RP Veltman D Machesky LM Actin-bundling proteins in cancer progression at a glance J Cell Sci 2012 125 5 1073 1079 10.1242/jcs.093799 22492983

[B42] Mori S Chang JT Andrechek ER Matsumura N Baba T Yao G Kim JW Gatza M Murphy S Nevin JR Anchorage-independent cell growth identifies tumors with metastatic potential Oncogene 2009 28 31 2796 2805 10.1038/onc.2009.139 19483725 PMC3008357

[B43] Takahashi M Furihata M Akimitsu N Watanabe M Kaul S Yumoto N Okada T A highly bone marrow metastatic murine breast cancer model established through in vivo selection exhibits enhanced anchorage-independent growth and cell migration mediated by ICAM-1 Clin Exp Metastasis 2008 25 5 517 529 10.1007/s10585-008-9163-5 18340424

[B44] Cifone MA Fidler IJ Correlation patterns of anchorage-independent growth with in vivo behavior of cells from a murine fibrosarcoma Proc Natl Acad Sci U S A 1980 77 2 1039 1043 10.1073/pnas.77.2.1039 6928659 PMC348419

[B45] Kuhlman PA Ellis J Critchley DR Bagshaw CR The kinetics of the interaction between the actin-binding domain of alpha-actinin and F-actin FEBS Lett 1994 339 297 301 10.1016/0014-5793(94)80434-6 8112470

